# *lytA* Quantitative PCR on Sputum and Nasopharyngeal Swab Samples for Detection of Pneumococcal Pneumonia among the Elderly

**DOI:** 10.1128/JCM.01231-17

**Published:** 2017-12-26

**Authors:** Annika Saukkoriipi, Arto A. Palmu, Thierry Pascal, Vincent Verlant, William P. Hausdorff, Jukka Jokinen

**Affiliations:** a Department of Public Health Solutions, National Institute for Health and Welfare, Oulu, Finland; b Department of Public Health Solutions, National Institute for Health and Welfare, Tampere, Finland; c GSK, Wavre, Belgium; d Department of Public Health Solutions, National Institute for Health and Welfare, Helsinki, Finland; Marquette University

**Keywords:** community-acquired pneumonia, nasopharynx, PCR, sputum, Streptococcus pneumoniae

## Abstract

Real-time quantitative PCR (qPCR) assay of sputum or nasopharyngeal specimens has shown promising results in the detection of pneumococcal community-acquired pneumonia (PncCAP). We applied qPCR for the autolysin gene (*lytA*) and compared sputum and nasopharyngeal swab (NPS) pneumococcal loads in elderly patients with community-acquired pneumonia (CAP), and specifically in patients with PncCAP, to those in patient groups with other respiratory diseases. We studied patients aged ≥65 years with radiologically confirmed CAP, clinical CAP not retrospectively radiologically confirmed, other acute respiratory infections, or stable chronic lung disease. Pneumococcal etiology of CAP was ascertained by using a combination of multiple diagnostic methods. We analyzed sputum and NPS specimens by *lytA* qPCR with 10^4^ pneumococcal genome equivalents (GE)/ml as a cutoff for positivity. Among PncCAP patients, *lytA* qPCR detected pneumococci in 94% of the sputum samples and in large quantities (mean, 6.82 ± 1.02 log_10_ GE/ml) but less frequently in NPS (44%) and in smaller quantities (5.55 ± 0.92 log_10_ GE/ml). In all other patient groups, ≤10% of the sputum samples and <5% of the NPS samples were *lytA* qPCR positive; but when they were positive, the sputum pneumococcal loads were similar to those in the PncCAP patients, suggesting a pneumococcal etiology in these patients. This was supported by other pneumococcal assay results. Overall, sputum *lytA* qPCR positivity was more common in PncCAP patients than in the other patient groups, but the quantitative results were mainly similar. NPS *lytA* qPCR was less sensitive than sputum *lytA* qPCR in detecting PncCAP.

## INTRODUCTION

Streptococcus pneumoniae is the most frequently detected bacterial etiologic agent in community-acquired pneumonia (CAP) (1), and the incidence of pneumococcal pneumonia in the elderly increases with age (2, 3). When conventional identification methods such as blood and sputum cultures are used, pneumococcal pneumonia remains underdiagnosed (4). Newer diagnostic methods such as pneumococcal urine antigen detection and PCR on respiratory specimens have consequently been applied to increase the sensitivity of case detection (5–8). A real-time quantitative PCR (qPCR) assay with the autolysin gene (*lytA*) as the target and applied to sputum (8, 9) or nasopharyngeal specimens (8–10) has shown promising results in the diagnosis of pneumococcal CAP (PncCAP). Here, we studied the genomic pneumococcal loads in sputum samples and nasopharyngeal swab (NPS) specimens from elderly patients with CAP, and specifically in patients with PncCAP (J. Jokinen et al., submitted for publication) (11), but also in patients with acute respiratory tract infections other than CAP and those with chronic lung disease (CLD), and compared the results between the groups.

(Data from this study were presented at the 10th International Symposium on Pneumococci and Pneumococcal Diseases, Glasgow, Scotland, 26 to 30 June 2016.)

## MATERIALS AND METHODS

### Study participants.

We studied noninstitutionalized patients ≥65 years old participating in a 2-year Finnish CAP epidemiological (FinCAP Epi) study in 2005 to 2007 (11). CAP was defined as any new opacity in a chest X-ray compatible with pneumonia in a symptomatic patient and confirmed retrospectively radiologically by at least two of three reviewers (two radiologists from Tampere University Hospital and one international reviewer). Clinical CAP cases that were not retrospectively radiologically confirmed were designated rejected CAP cases. Patients with an acute respiratory infection (ARI) and no radiological signs of pneumonia and no diagnosis of pneumonia in the 30 days preceding and the 30 days following enrollment were enrolled as one comparison group, and patients with a CLD (International Classification of Diseases 10th revision discharge diagnosis of chronic bronchitis, lung emphysema, or chronic obstructive pulmonary disease) were enrolled as another comparison group. The CLD patients underwent spirometry and were in stable condition without concurrent exacerbation. Informed consent was obtained from all subjects or their next of kin/guardians. The study protocol was approved by the ethical review board of the Pirkanmaa Hospital District, the Tampere City Health Center, and the institutional review board at the National Institute for Health and Welfare (THL).

### Microbiological assessment.

The collection and processing of study samples, including blood specimens for aerobic and anaerobic blood cultures and serology, sputum, NPS, and urine, as well as the assays carried out to detect pneumococci, have been described in detail previously (11). The sputum samples were spontaneously produced or induced with a NaCl nebulizer. Sputum quality was assessed by microscopy after Gram staining and was rated high (high-quality [HQ] sputum) if the ratio of leukocytes to epithelial cells was >1. This criterion for HQ sputum differs from that described by Murray and Washington (12) but has been used previously (13). However, all sputum samples were processed and assayed as planned, irrespective of their quality. Part of each sputum sample was stored for viral analyses, and the rest was treated with Sputolysin (dithiothreitol). Part of the processed sputum was stored at −70°C for DNA extraction and real-time qPCR, and another part was plated undiluted and diluted 1/100 on sheep blood agar, chocolate agar, and blood agar plates with gentamicin. Alpha-hemolytic colonies suspected to be S. pneumoniae were identified primarily by using optochin sensitivity and, if needed, by using bile solubility and the quellung reaction with pneumococcal omniserum. NPS samples were collected with a calcium alginate swab in STGG (skim milk, tryptone, glucose, and glycerol) medium and stored at −70°C. DNA was extracted from sputum and NPS samples with the MagNA Pure LC DNA isolation kit III (bacteria, fungi) and a MagNA Pure LC instrument (Roche Diagnostics, Germany). Real-time *lytA* qPCR was carried out with the LightCycler 2.0 instrument (Roche Diagnostics) and a previously described method (14) in which a 173-bp fragment of the *lytA* gene is amplified and detected and a melting curve program is used to check the specificity of probe binding. The sample volume in each reaction mixture was 5 μl, and each qPCR run included a standard containing 500 pneumococcal genome equivalents (GE). No internal process control was used. The data were analyzed with LightCycler software version LCS4 4.0.5.415 (Roche Diagnostics). An external standard curve created by amplifying four replicates of six serial dilutions of purified pneumococcal DNA (5 × 10^5^, 5 × 10^4^, 5 × 10^3^, 5 × 10^2^, 5 × 10^1^, and 5 GE) was used for quantification. Ten thousand GE/ml of sample (50 GE/qPCR) was used as a cutoff for positivity for both specimen types, as this was the lowest concentration that could be detected in at least 95% of the assays when dilution series of four independently extracted negative patient samples with a known amount of added pneumococcal DNA were analyzed in six replicates as part of the validation of the *lytA* qPCR method carried out separately for both specimen types.

During the first year of this study, clinical CAP patients were assayed for certain viruses, including the influenza A and B viruses (sputum PCR and serology), respiratory syncytial virus (RSV; sputum PCR and serology), and parainfluenza virus type 1 (PIV1), PIV2, and PIV3 (sputum PCR) (11).

### Criteria for PncCAP.

We used our previously published definition of PncCAP (11), which was constructed by utilizing latent class analysis (LCA) to determine true disease status on the basis of imperfect tests and is presented in detail elsewhere (Jokinen et al., submitted). PncCAP was defined as (i) encapsulated pneumococci cultured from blood, (ii) encapsulated pneumococci cultured from HQ sputum, or (iii) at least two of the following: (i) a ≥2-fold increase in serum antipneumococcal surface adhesin A (PsaA) and/or anti-choline-binding protein A (CbpA) antibodies, (ii) a positive pneumococcal urine antigen test, or (iii) detection of pneumococci in sputum of any quality or NPS by culture (encapsulated) or *lytA* qPCR (11).

### Data analysis.

Pearson's chi-square test or Fisher's exact test, where appropriate, was used for the comparison of categorical variables. The qPCR results were log_10_ transformed and compared between and within the patient groups. The Student *t* test was used for comparisons of means, and the correlation of pneumococcal genomic loads in sputum and NPS samples was calculated by using Pearson's correlation coefficient. A *P* value of <0.05 was considered statistically significant. Statistical analyses were performed with SPSS version 22 (SPSS Inc., Chicago, IL).

The CURB-65 score, a measure of severity of pneumonia, was calculated as described by Lim et al. (15).

## RESULTS

**FIG 1:**
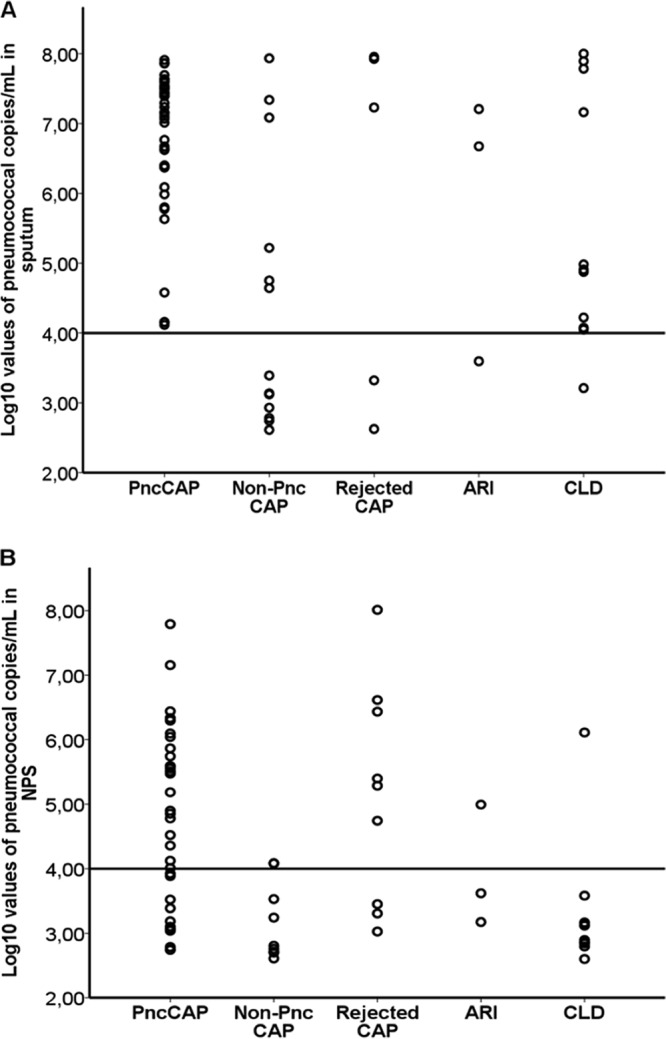
Log_10_-transformed results of *lytA* qPCR assays of sputum (A) and NPS (B) samples from patients with PncCAP, non-PncCAP, clinical CAP that was not radiologically confirmed (rejected CAP), ARI, and CLD. All results greater than zero are included. The horizontal line shows the cutoff for positivity (10^4^ GE/ml) that was used in this study.

When 10^4^ GE/ml was used as the cutoff for positivity, pneumococci were detected by *lytA* qPCR in sputum samples from 44/47 (94%) PncCAP cases, 10/103 (10%) CLD cases, and <5% of the cases in the other groups. NPS specimens were positive for *lytA* in 23/52 (44%) PncCAP cases and in <5% of those in all other groups (Table 1). There were 0 PncCAP, 7 non-PncCAP, 2 rejected CAP, 1 ARI, and 1 CLD cases with a *lytA* qPCR result below the cutoff but greater than zero in sputum and 12, 7, 3, 2, and 11 in NPS, respectively. The log_10_-transformed *lytA* qPCR results of the sputum and NPS samples with a result greater than zero among the patient groups are shown in Fig. 1A and B, respectively. The samples with a quantitative result below the cutoff were considered negative and were not included in the following analyses.

**TABLE 1:** Percentage of positive cases^*e*^ and pneumococcal loads in the different patient groups assayed by using *lytA* qPCR

Patient group	Sputum				NPS			
	*n*	No. (%) *lytA* qPCR positive	Mean log_10_ GE/ml ± SD^*a*^	*P* value^*b*^	*n*	No. (%) *lytA* qPCR positive	Mean log_10_ GE/ml ± SD^*a*^	*P* value^*b*^
Confirmed CAP	224	50 (22)	6.74 ± 1.09		306	25 (8)	5.44 ± 0.97	
PncCAP^*c*^	47	44 (94)	6.82 ± 1.02		52	23 (44)	5.55 ± 0.92	
Non-PncCAP	177	6 (3)^*d*^	6.16 ± 1.45	0.33	254	2 (1)^*d*^	4.08 ± 0.00	0.04
Rejected CAP	106	3 (3)^*d*^	7.70 ± 0.41	0.15	157	6 (4)^*d*^	6.08 ± 1.19	0.25
ARI	65	2 (3)^*d*^	6.94 ± 0.38	0.87	80	1 (1)^*d*^	4.99	
CLD	103	10 (10)^*d*^	5.80 ± 1.70	0.09	121	1 (1)^*d*^	6.11	

a Only *lytA* qPCR positive cases included.

b Quantitative results compared to PncCAP.

c PncCAP was defined as (i) encapsulated pneumococci cultured from blood, (ii) encapsulated pneumococci cultured from HQ sputum (a leukocyte/epithelial cell ratio of >1), or (iii) at least two of the following: (i) a ≥2-fold increase in serum anti-PsaA and/or anti-CbpA antibodies, (ii) a positive pneumococcal urine antigen test, (iii) detection of pneumococci from sputum of any quality or NPS by culture (encapsulated) or *lytA* qPCR.

d *P* < 0.001 compared to PncCAP.

e The cutoff for positivity was 10^4^ GE/ml.

**FIG 2:**
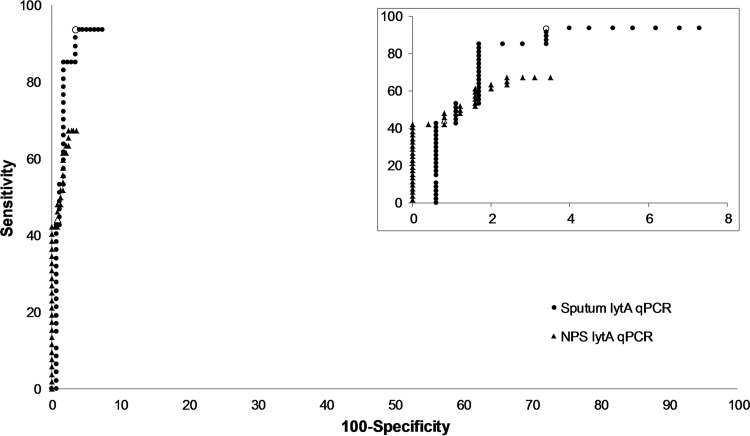
Diagnostic performance (sensitivity versus 100 − specificity) of *lytA* qPCR applied to sputum and NPS samples for detection of PncCAP among patients with CAP and the sensitivity and specificity of *lytA* qPCR at the cutoff for positivity (10^4^ GE/ml) for sputum (○), and NPS (Δ). The inset contains the same data but with a smaller *x*-axis scale.

The diagnostic performance of *lytA* qPCR for the detection of PncCAP among CAP patients is presented in Fig. 2. At the predefined cutoff of 10^4^ GE/ml of sample, the sensitivity and specificity of the *lytA* qPCR test were 94 and 97% for sputum samples and 44 and 99% for NPS samples, respectively. The criteria for PncCAP (Jokinen et al., submitted) (11) included *lytA* qPCR from sputum or NPS but only in combination with a ≥2-fold increase in paired serum anti-PsaA and/or anti-CbpA antibodies or a positive pneumococcal urine antigen test. There were four cases in which the PncCAP diagnosis depended on a positive sputum *lytA* qPCR result, and in three of these, pneumococcal serology or the urine antigen test was missing. When *lytA* qPCR positivity was left out of the criteria for PncCAP, the sensitivity and specificity of the *lytA* qPCR test with sputum were similar (93 and 94%, respectively). There were no cases in which the diagnosis depended on a positive NPS *lytA* qPCR result.

While only ≤10% of the non-PncCAP, rejected CAP, ARI, or CLD cases were positive for pneumococcus by *lytA* qPCR, when positive their mean pneumococcal load (± standard deviation) in sputum did not differ statistically significantly from that of the PncCAP cases (Table 1). This was true also when only HQ sputum samples were included in the analysis (data not shown). The five patients with ARI or rejected CAP that had a positive *lytA* qPCR result and a high pneumococcal density in sputum (Table 1) had encapsulated pneumococci cultured from their sputum samples, which all were of high quality. Two of these patients also had a positive urine antigen test result. This suggests a pneumococcal etiology in these five patients, as they fulfilled the etiological case definition but not the radiological CAP definition.

**FIG 3:**
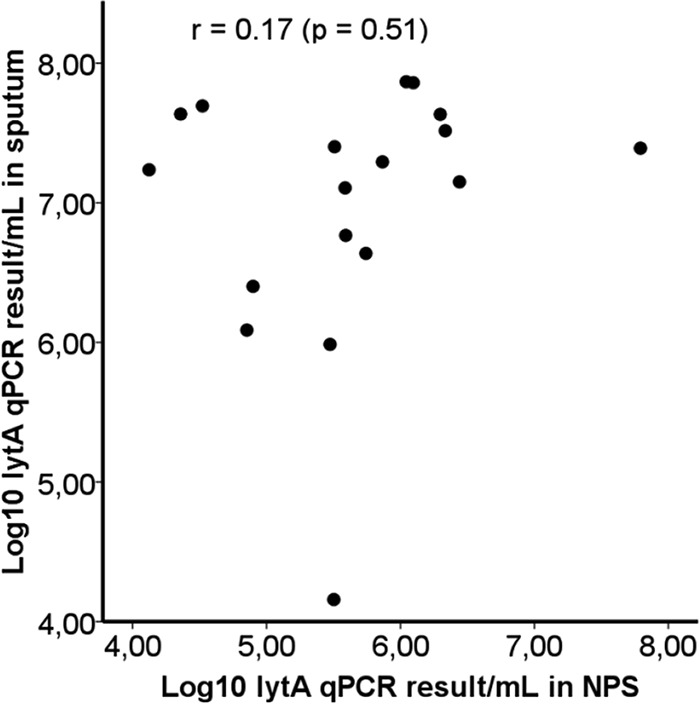
Correlation of sputum *lytA* qPCR results with NPS *lytA* qPCR results among patients with PncCAP and a positive *lytA* qPCR result with both sputum and NPS samples (*n* = 18).

In the subgroup of patients with positive *lytA* qPCR results (≥10^4^ GE/ml) with both sputum and NPS, the *lytA* qPCR results showed no correlation between sputum and NPS pneumococcal genomic loads (*n* = 23, Pearson's correlation *r* = 0.19, and *P* = 0.39 for all cases; *n* = 19, *r* = 0.14, and *P* = 0.56 for CAP cases; *n* = 18, *r* = 0.17, and *P* = 0.51 for PncCAP cases) (Fig. 3).

In the patients with a positive sputum *lytA* qPCR result, the mean pneumococcal genomic copy numbers were higher in HQ sputum than in low-quality (LQ) sputum among all cases (*n* = 53, 6.78 ± 1.15 versus *n* = 11, 5.91 ± 1.35 log_10_ GE/ml, *P* = 0.03); however, among CAP cases only (Table 2) or among PncCAP cases only (*n* = 38, 6.88 ± 0.96 versus *n* = 5, 6.15 ± 1.37 log_10_ GE/ml, *P* = 0.13), the difference was not statistically significant.

**TABLE 2:** Factors and their association with sputum pneumococcal loads among CAP cases assayed by *lytA* qPCR

Factor	Yes			No			*P* value^*b*^	*P* value^*c*^
	*n*	No. (%) *lytA* qPCR positive	Mean log_10_ GE/ml ± SD^*a*^	*n*	No. (%) *lytA* qPCR positive	Mean log_10_ GE/ml ± SD^*a*^		
Male	121	31 (26)	6.50 ± 1.25	103	19 (18)	7.13 ± 0.59	0.20	**0.02**
Current smoker	27	8 (30)	6.69 ± 1.33	185	38 (21)	6.70 ± 1.08	0.28	0.98
HQ sputum^*d*^	169	40 (24)	6.86 ± 0.98	53	9 (17)	6.13 ± 1.40	0.31	0.17
Encapsulated pneumococci cultured from sputum	40	37 (93)	7.22 ± 0.57	184	13 (7)	5.38 ± 1.07	**<0.001**	**<0.001**
Influenza A virus-positive sputum PCR and/or serology	7	2 (29)	6.24 ± 0.62	96	21 (22)	6.80 ± 1.09	0.65	0.49
Influenza B virus-positive sputum PCR and/or serology	0	NA	NA	101	23 (23)	6.75 ± 1.06	NA	NA
RSV-positive sputum PCR and/or serology	14	4 (29)	7.59 ± 0.26	88	19 (22)	6.58 ± 1.09	0.51	**0.002**
PIV1, -2, or -3-positive sputum PCR	7	4 (57)	7.27 ± 0.64	104	21 (20)	6.69 ± 1.07	**0.04**	0.31
Any virus^*e*^	28	10 (36)	7.19 ± 0.69	76	13 (17)	6.42 ± 1.20	**0.04**	0.07
Antibiotic use at visit only	41	13 (32)	5.75 ± 1.21	138^*f*^	34 (25)	7.06 ± 0.82	0.37	**0.002**
Antibiotic use within 2 wk before visit	39	3 (8)	7.47 ± 0.07	138^*f*^	34 (25)	7.06 ± 0.82	**0.03**	0.40
Hospitalization	184	38 (21)	6.72 ± 1.15	40	12 (30)	6.81 ± 0.90	0.20	0.81
CURB-65 score of 3, 4, or 5^*g*^	50	12 (24)	6.92 ± 0.78	167	37 (22)	6.66 ± 1.18	0.78	0.47

a Only qPCR-positive cases included.

b Proportions of *lytA* qPCR-positive samples compared with the Pearson chi-square test or Fisher exact test. Statistically significant values are in boldface.

c Genomic loads compared with the Student *t* test. Statistically significant values are in boldface.

d More than one leukocyte per epithelial cell.

e Positive sputum PCR for PIV1, -2, or -3 or positive sputum PCR or serology for influenza A virus, influenza B virus, or RSV.

f No use of antibiotics at visit or within 2 weeks before visit.

g CURB-65: confusion, urea concentration of >7 mmol/liter, respiratory rate of ≥30/min, low blood pressure (systolic <90 mm Hg or diastolic ≤60 mm Hg), age of ≥65 years (15).

Pneumococci were cultured from 50 (22%) CAP cases' sputum and 37 (12%) CAP cases' NPS samples, and the cultured pneumococci were encapsulated in 40 (80%) and 32 (86%) of the cases, respectively; the *lytA* qPCR result was positive in 37 (93%) and 22 (69%) of these, respectively, and negative in all cases with unencapsulated pneumococci cultured from the same sample type. Among the CAP cases with a negative pneumococcal culture, the *lytA* qPCR was positive in 13 (7%) and 3 (1%) cases with sputum and NPS samples, respectively. In the patients with a positive sputum *lytA* qPCR result, the mean pneumococcal genomic load in sputum was greater among the cases with pneumococci (encapsulated) cultured from sputum than among those with no pneumococci cultured from sputum (Table 2). The same was true of NPS samples (5.59 ± 0.93 versus 4.33 ± 0.49 log_10_ GE/ml, *P* = 0.03).

The *lytA* qPCR positivity and quantitative results of those with a positive *lytA* qPCR result were also explored for sputum in some selected subgroups of CAP patients (Table 2). The CAP patients with any respiratory viral coinfection (influenza A or B virus, RSV, PIV1, PIV2, or PIV3) were more often *lytA* qPCR positive and had a slightly higher pneumococcal genomic load than those with no viruses detected, although the difference in the quantitative result was not statistically significant. However, among the patients with RSV, the mean pneumococcal load was significantly greater (Table 2). The pneumococcal genomic load of the CAP patients with a CURB-65 score of 3 to 5 did not differ from that of the patients with a CURB-65 score 1 or 2, and the pneumococcal genomic loads in hospitalized and nonhospitalized patients were similar (Table 2). The CAP patients who had received antibiotics at their acute-phase visit before sputum sampling had a lower mean sputum pneumococcal genomic load than those who were not exposed to antibiotics at the visit or within 2 weeks before the visit (Table 2). The analyses presented in Table 2 were also conducted by including only the CAP cases with no antimicrobial exposure in the 2 weeks before the visit or at the visit (*n* = 138; *lytA* qPCR positive *n* = 34). The results were similar to those in Table 2, except that there was no difference between the pneumococcal loads of females and males when only patients with no antimicrobial use were included (data not shown).

## DISCUSSION

In the present study of elderly patients, pneumococci were detected by *lytA* qPCR in the majority of the PncCAP patients' sputum samples and less frequently and in smaller quantities in NPS. In all other patient groups, the prevalence of pneumococci by *lytA* qPCR was low. However, when the test was positive, a high pneumococcal density was observed in the sputum of ARI and rejected CAP patients, suggesting a pneumococcal etiology in these patients.

The diagnostic performance of the *lytA* qPCR test was studied by comparing it to the criteria used for PncCAP in the FinCAP Epi study (11). The criteria were derived by LCA with a focus on an optimal case definition for vaccine trial purposes (Jokinen et al., submitted), and they included *lytA* qPCR positivity with sputum or NPS in combination with a positive urine antigen test result or a ≥2-fold increase in paired serum anti-PsaA and/or anti-CbpA antibodies. Therefore, comparison of *lytA* qPCR test performance against these criteria may not be optimal for the determination of *lytA* qPCR sensitivity. On the basis of the LCA model, the sensitivity of *lytA* qPCR with sputum was 90% (Jokinen et al., submitted), which is likely to be closer to the real sensitivity of the test. Also, the criterion for HQ sputum used in our definition of PncCAP (11) and in the present study differs from that described by Murray and Washington (12). We have previously evaluated the significance of sputum quality in sputum culture for the diagnosis of PncCAP by using more stringent criteria for HQ sputum (a leukocyte/epithelial cell ratio of >5 and ≤2.5 epithelial cells/field) and found that cultures positive for encapsulated pneumococci from HQ and LQ sputum samples showed a similar concordance with other pneumococcal diagnostic tests if the other test was positive (16). If the other pneumococcal diagnostic test was negative, encapsulated pneumococci were isolated from LQ sputum samples less often than from HQ sputum samples (16). As our previous results did not support the concept that encapsulated pneumococci cultured from an LQ sputum sample would more probably be a false-positive indicator of pneumococcal etiology in CAP than the same finding in an HQ sample (16), we applied a less stringent criterion for HQ sputum in the PncCAP definition (Jokinen et al., submitted) (11) in the present study.

In a recent study, Strålin et al. (8) used a *lytA* qPCR assay and reported a prevalence of positive results with sputum samples (94% with a cutoff of 10^5^ copies/ml and 97% with a cutoff of 10^4^ copies/ml) from adult PncCAP patients (median age, 71 years) similar to that in the present study. They also reported a similar mean sputum pneumococcal load (6.71 ± 1.01 log_10_ copies/ml) in their qPCR-positive cases. In nasopharyngeal aspirates (NPA), they detected positive results among PncCAP patients by using their *lytA* qPCR assay more often (62% with a cutoff of 10^4^ copies/ml) than we did in NPS specimens, but the mean nasopharyngeal pneumococcal load (5.35 ± 1.69 log_10_ copies/ml) among the qPCR-positive cases was similar to that reported here (8). NPA and NPS sampling methods for detecting pneumococcal pharyngeal carriage by culture have been compared in children by Rapola et al. (17), and they found no significant difference in the rate of pneumococcus isolation between NPA and NPS by culture. Among the patients with non-PncCAP and a positive *lytA* qPCR result, we detected a greater mean pneumococcal load in sputum than Strålin et al. did (8). In agreement with their results, we found no correlation between the pneumococcal genomic copy numbers in sputum and nasopharyngeal specimens. However, Albrich et al. (9) previously noted a good correlation between the genomic pneumococcal loads in sputum and NPS from HIV-infected adults with CAP. The studies of Strålin et al. (8) and Albrich et al. (9) did not include patients with respiratory tract infections other than CAP.

The mean pneumococcal load detected by qPCR was greater in patients with a positive sputum culture (encapsulated pneumococci) than in patients with no pneumococci cultured. This is in accordance with previous studies where greater pneumococcal genomic loads have been observed in culture-positive sputum specimens (5, 9, 18). Gadsby et al. (5), however, noted that their culture-negative group was more frequently exposed to antibiotics, which was also associated with lower bacterial loads. Werno et al. (18) did not find any significant effect of antibiotic use on genomic pneumococcal loads, even though they detected pneumococci less often in the sputum of patients exposed to antibiotics prior to admission. We have previously studied the effect of antibiotic use on pneumococcal diagnostic tests with the same study population as here (19) and found that antibiotic use within 2 weeks before the acute-phase visit, and specifically when still ongoing at enrollment, but not antibiotic use at the visit, was associated with lower sputum *lytA* qPCR positivity. In the present study, patients who had received antibiotics at the acute-phase visit, but not those who had gotten antibiotics within 2 weeks before the visit, had a significantly lower mean pneumococcal genomic load in their sputum than those with no antibiotic use.

In all CAP cases in which unencapsulated pneumococci were cultured, the *lytA* qPCR result with the same sample type was negative. This implies that the unencapsulated cultured isolates were, in fact, not true pneumococci and they were disregarded in the further analyses.

It is well known that respiratory virus infections play a role in respiratory bacterial infections (20). Respiratory virus coinfection has also been associated with greater nasopharyngeal pneumococcal loads in children with CAP (21) and in ARI patients with a high HIV prevalence (22). Alpkvist et al. (23), however, found no association between viral coinfection and greater nasopharyngeal pneumococcal loads in adult patients. In the present study, virus infection was detected by sputum PCR and/or serology and CAP patients with a respiratory viral coinfection had a slightly, although not statistically significantly, greater sputum pneumococcal genomic load than CAP patients with no viruses detected. Among the CAP patients with RSV, the pneumococcal load was, however, significantly greater. Interestingly, in mice, RSV infection has been shown to decrease the clearance of pneumococci from the lungs and increase pulmonary inflammation (24).

A high nasopharyngeal pneumococcal density has been found to be associated with greater disease severity (23, 25). However, when the sputum pneumococcal load and disease severity were compared by Werno et al. (18), no association was found except for those patients who were previous and current smokers; they were more often in a higher pneumonia severity index risk class when the pneumococcal load in sputum was >10^3^ CFU/ml. We analyzed the pneumococcal loads in the sputum samples of CAP patients, their CURB-65 scores, and their hospitalization status for associations and found none.

The present study confirms the results of the study of Strålin et al. (8), which identified the *lytA* qPCR assay of sputum as a useful method for the diagnosis of PncCAP. In contrast to the study of Albrich et al. (9), the *lytA* qPCR assay of NPS did not perform as well as the *lytA* qPCR assay of sputum samples in the present study of elderly persons. The quantitative result obtained with NPS may be less reliable because of variations in the quantity of the actual sample and in the recovery of pneumococci from STGG. The *lytA* qPCR assay is a more rapid method than culture. We did not try to identify optimal cutoff values for the diagnosis of PncCAP by using *lytA* qPCR but used a predefined cutoff that was the lowest concentration that could be consistently detected. The strengths of the present study are that it was a prospective follow-up study with systematic data collection and that it included different patient groups. Thus, we had qPCR results available also for patient groups with respiratory tract infections other than CAP or a CLD. However, a limitation of this study was the low number of patients, especially in the PncCAP group. In addition, only a few patients in the present study had pneumococcal bacteremia. A potential limitation of the *lytA* qPCR assay is that *lytA* has been found in some nonpneumococcal streptococcal isolates of the Streptococcus mitis group (26, 27). Thus, the *lytA* qPCR assay potentially overestimates the pneumococcus as the etiological agent of CAP. In the present study, 3% of the non-PncCAP cases' sputum samples and 1% of their NPS samples were positive by *lytA* qPCR assay but the etiological agent in these was not determined.

In conclusion, pneumococci were detected by *lytA* qPCR in the majority of the PncCAP patients' sputum samples when 10^4^ GE/ml was used as the cutoff for positivity. In all other patient groups, the prevalence of pneumococci by *lytA* qPCR was low. Sputum was superior to NPS as a sample type in the detection of PncCAP by *lytA* qPCR, and *lytA* qPCR analysis of sputum continues to be a promising diagnostic tool in the detection of pneumococcal etiology in CAP. A trend toward greater pneumococcal loads in sputum samples from CAP patients with a viral coinfection, particularly RSV, was seen.
